# Editorial Summary of the Special Issue: Healthcare Practice in Community

**DOI:** 10.3390/healthcare14172878

**Published:** 2026-09-07

**Authors:** Florence M. F. Wong, Wai Keung Leung

**Affiliations:** 1 School of Nursing, Tung Wah College, Hong Kong; 2 Faculty of Dentistry, University of Hong Kong, Pok Fu Lam, Hong Kong; ewkleung@hku.hk

We are pleased to present this special issue, “Healthcare Practice in Community”, which features 11 insightful publications that reflect the current landscape of community practice and scientific evidence. These 11 papers include 7 original research articles, 1 narrative review, 1 systematic literature review, 1 opinion paper, and 1 patient narrative/personal experience piece, covering 11 countries and regions to examine community-based healthcare practice with challenges and innovations from a global perspective.

The original research articles cover four thematic areas. (1) Caregiver support is addressed by two qualitative studies: one reveals unmet needs and systemic transition gaps for families of individuals with severe developmental disabilities in Northern Ireland, while another validates virtual Communities of Practice as a platform for emotional and professional support among dementia caregivers. (2) Older adult care features two validation studies: (a) a Taiwanese palliative care screening tool for home-based patients and (b) an oral health assessment instrument for long-term care workers in Hong Kong. (3) Workforce development is explored through a Spanish study identifying poor health behaviors and low self-concept among health sciences students, and a survey of community pharmacists in Egypt and Iraq showing satisfactory attitudes but insufficient knowledge about proton pump inhibitor use. (4) Health access and equity are examined via GIS mapping of specialist shortages across Texas, revealing severe rural deficits in cardiology, pulmonology, and endocrinology.

The review papers have offered important conceptual contributions. The narrative review examined knowledge translation in Saudi Arabia’s primary care and community health sectors, contextualizing its findings within the Saudi Vision 2030 reforms and emphasizing the need for stakeholder engagement and community-based participatory research to bridge the evidence-practice gap. The systematic literature review evaluated quality indicators for home-based community elderly care in China, concluding that existing frameworks were fragmented, lacked cultural specificity, and overemphasized structural measures while neglecting the outcomes.

The two non-empirical contributions included an opinion piece proposing culturally tailored strategies to increase ethnic minority participation in cardiovascular research, and a patient narrative that highlighted the critical role of advocacy and community engagement in amyotrophic lateral sclerosis care.

Overall, these 11 papers have underscored that effective community healthcare hinges on addressing workforce training gaps, geographic and ethnic disparities, fragmented quality measurement, and insufficient caregiver support. The issue presents actionable pathways, including validated assessment tools, virtual learning platforms, GIS-informed planning, and participatory research, while affirming that sustainable progress requires integrated action across policy, education, technology, and community engagement, with special attention to vulnerable populations and their care providers.

